# To Text or Not to Text: Electronic Message Intervention to Improve Treatment Adherence Versus Matched Historical Controls

**DOI:** 10.2196/11720

**Published:** 2019-04-09

**Authors:** Marily A Oppezzo, Michael V Stanton, Ariadna Garcia, Joseph Rigdon, Jae R Berman, Christopher D Gardner

**Affiliations:** 1 Stanford Prevention Research Center Stanford University School of Medicine Stanford, CA United States; 2 Department of Health Sciences California State University, East Bay Hayward, CA United States; 3 Quantitative Sciences Unit Stanford University School of Medicine Stanford, CA United States

**Keywords:** treatment adherence, intervention, short message service, mobile health, propensity score

## Abstract

**Background:**

Ensuring treatment adherence is important for the internal validity of clinical trials. In intervention studies where touch points decrease over time, there is even more of an adherence challenge. Trials with multiple cohorts offer an opportunity to innovate on ways to increase treatment adherence without compromising the integrity of the study design, and previous cohorts can serve as historical controls. Electronically delivered nudges offer low-cost opportunities to increase treatment adherence.

**Objective:**

This study aimed to evaluate the effectiveness of electronic messages (e-messages) on treatment adherence to the last cohort of a parent weight loss intervention during the second half of a year-long trial, when intervention checkpoint frequency decreases. Treatment adherence is measured by intervention class attendance and adherence to the intervention diet.

**Methods:**

All participants in the last cohort (cohort 5, n=128) of a large randomized weight loss study were offered an e-message intervention to improve participant adherence during the last 6 months of a 1-year weight loss program. Overall, 3 to 4 electronic weekly messages asked participants about intervention diet adherence. A propensity score model was estimated using 97 participants who opted to receive e-messages and 31 who declined in cohort 5 and used to pair match cohort 5 e-message participants to a historical control group from cohorts 1 to 4. Moreover, 88 participants had complete data, yielding 176 participants in the final analyses. After matching, intervention and matched control groups were compared on (1) proportion of class attendance between the 6 and 12 month study endpoints, (2) diet adherence, as measured by total carbohydrate grams for low-carbohydrate (LC) and total fat grams for low-fat (LF) diets at 12 months, and (3) weight change from 6 to 12 months. The dose-response relationship between the proportion of text messages responded to and the 3 outcomes was also investigated.

**Results:**

Compared with matched controls, receiving e-messages had no effect on (1) treatment adherence; class attendance after 6 months +4.6% (95% CI −4.43 to 13.68, *P*=.31), (2) adherence; LC −2.5 g carbohydrate, 95% CI −29.9 to 24.8, *P*=.85; LF +6.2 g fat, 95% CI −4.1 to 17.0, *P*=.26); or on (3) the secondary outcome of weight change in the last 6 months; +0.3 kg (95% CI −1.0 to 1.5, *P*=.68). There was a positive significant response correlation between the percentage of messages to which participants responded and class attendance (*r*=.45, *P*<.001).

**Conclusions:**

Although this e-message intervention did not improve treatment adherence, future studies can learn from this pilot and may incorporate more variety in the prompts and more interaction to promote more effective user engagement. Uniquely, this study demonstrated the potential for innovating within a multicohort trial using propensity score–matched historical control subjects.

**Trial Registration:**

ClinicalTrials.gov NCT01826591; https://clinicaltrials.gov/ct2/show/NCT01826591

**International Registered Report Identifier (IRRID):**

RR2-10.1016/j.cct.2016.12.021

## Introduction

### Background

An important challenge of behavioral interventions is treatment adherence. Treatment adherence strategies increase participants’ enactment of the intervention delivered, thereby increasing the internal validity of the results and accuracy of conclusions drawn from the study [1,2]. Weight loss diet interventions often show high recidivism, with adherence to the treatment an inherent challenge, and often built into the intervention itself [3-6]. With adherence being highly variable in diet studies, it becomes difficult to evaluate the effectiveness of diet types. Applying treatment adherence strategies to all treatment arms, or diet groups, would extend participant contact and enhance the strength of study conclusions. Furthermore, in large trials with multiple cohorts or staggered enrollment, there is an opportunity to utilize lessons learned from early enrollees to design protocol modifications to increase treatment adherence in later enrollees. This would be the case for the Diet Intervention Examining The Factors Interacting with Treatment Success (DIETFITS) study, which contrasted healthy low-carbohydrate (LC) versus healthy low-fat (LF) diets for a sample of 609 generally healthy, free-living adults with body mass index 28 to 40 kg/m^2^ [7].

### Objectives

This paper highlights the deployment of a treatment adherence enhancement for the fifth cohort of a large 5-cohort, weight loss trial testing the differential effects of adhering to randomly assigned, high-quality, LC or LF diets. The novelty of this paper is three-fold: first, our intervention demonstrates dynamic trial-design potential. It was developed to increase treatment adherence and was a response to feedback from the previous 4 cohorts from months 7 to 12 of the 12-month protocol. Our intervention was then deployed to *both* diet treatment arms of the parent study in cohort 5. Second, the evaluation of the effectiveness of our intervention capitalized on the preceding 4 cohorts to provide historical, matched control participants. Third, our intervention targeted *adherence to* the treatment (ie, in-person class attendance and adherence to the diet of either LC or LF content), *not* the intended outcome of the treatment (ie, weight loss). We explored weight loss only as a secondary outcome.

The National Institutes of Health’s Behavior Change Consortium’s Treatment Fidelity Workgroup outlined goals for treatment fidelity strategies across 5 research process phases [1]. Our intervention was designed to increase the *treatment skills enactment* phase of the larger DIETFITS trial. We define the treatment skills as attending the classes for their diet treatment (class attendance) and adhering to their assigned diet (diet adherence). Although our intervention is under the NIH Behavior Change Consortium’s umbrella of treatment fidelity, we will refer to it as treatment adherence in this paper.

The DIETFITS intervention gave both LC and LF groups weekly (8 classes), biweekly (5 classes), every third week (4 classes), and monthly (5 classes over months 7-12) education classes designed to improve participant adherence to the highest-quality version of their assigned diet. Postintervention focus groups and surveys from the first 4 cohorts of the DIETFITS trial indicated that participants desired more accountability during months 7 to 12, when face-to-face study contact substantially decreased. In addition, objective data from the first 4 cohorts showed a decrease in class attendance during months 7 to 12, averaging 46.8% compared with 73.8% in months 1 to 6. Diet adherence in the DIETFITS study had the broad guideline of reaching as low as the participants could go in carbohydrate or fat grams during the first 8 weeks and then titrating slowly back up to find the lowest amount they could maintain for the study duration. Adherence data, in the form of 24-hour recalls, were collected via phone at 4 major data collection time points during the DIETFITS trial. Similar to other studies, recidivism in diet studies tends to start after participants have lost significant amounts of weight and are having difficulty in maintaining behavioral changes [8,9], commonly 6 months. For cohorts 1 to 4 in DIETFITS, at 6 months, the healthy LC diet averaged 111.6 g of carbohydrates versus 128.0 g at 12 months, and the healthy LF diet averaged 50.0 g of fat at 6 months versus 56.1 g at 12 months. This average increase in grams of carbohydrates or fat during the second 6 months shows slight recidivism.

In response to participants’ requests and for the purpose of minimizing recidivism and maximizing adherence, we sought to increase treatment skills enactment during months 7 to 12 for cohort 5, specifically increasing class attendance and adherence to their diet. Extended contact after the initial intervention contact points have decreased has been shown to support long-term diet behavior change [10]. However, extended contact via face to face is costly and time-consuming. Our treatment adherence enhancement intervention (henceforth referred to as the e-message intervention) utilized short message service (SMS) text messages or email to enhance adherence. Electronically delivered messages are convenient, cost-effective, asynchronous (ie, can be read by participants at times that suits the individual) delivery channels for behavior change intervention touches and do not require labor-intensive face-to-face contact [11]. Using the internet to deliver periodic prompts to a behavior change method has been effective in 11 of the 19 reviewed studies [12], and another review article found that 13 of the 14 studies reviewed SMS-delivered interventions with positive, short-term effects on behavior change [11]. One weight loss study delivered 4 times daily SMS text messages with tips, self-monitoring reminders, and motivational messages and found that although message delivery was not a sufficient stand-alone intervention compared with a control group for significant weight loss, participants who responded to a greater proportion of SMS text messages tended to have the greatest weight loss at 6 and 12 months [13]. Another 4-month trial found significant weight loss in a 2- to 5- times daily personalized SMS text message intervention compared with a control group [14]. Although there is some evidence of success when using mobile technology to deliver interventions, less research has been conducted on using SMS text messages to extend intervention contacts or enhance treatment adherence [2,15]. One recent trial showed an average of 8 SMS text messages every 2 weeks as an intervention contact extension–supported attenuation of weight gain compared with those without the contact extension [16]. Our work builds on this success, using SMS text messages or email as a low-cost, adjunct strategy to extend contact when face-to-face contact substantially decreases. Distinctly, our e-message contacts target enhancing treatment adherence.

This study is an innovative approach to modifying an existing randomized controlled trial (RCT), after it had begun, to increase treatment adherence. In response to the first 4 RCT cohorts’ request for more accountability in the latter half of their treatment when experimenter interaction decreased, we created an e-message intervention and applied it to both arms of the RCT for cohort 5. Our e-message intervention was designed to increase all participants’ adherence to the treatment assigned, in this case, a diet plan. Treatment adherence was measured by class attendance and adherence to the diet assigned. Weight loss was a secondary outcome. We used propensity score matching to historical controls in the previous 4 cohorts.

## Methods

### Study Participants

#### Parent Trial

The DIETFITS trial is an institutional review board-approved large, randomized, weight loss study in which 609 participants were randomly assigned to either a (LC) or (LF) diet, with 16 classes spread throughout months 1 to 6 and 6 monthly booster classes during months 7 to 12. The primary dietary goal for both diet groups was to “go as low as you can go, and maintain” for carbohydrates in the LC group and for fat in the LF group; there was no set target number of grams or percent of calories to reach; therefore, the lower the grams of carbohydrate or fat achieved, the more adherent this was considered [17]. The trial was split into 5 cohorts over a 3-year period, with the first cohort (cohort 1) beginning in April 2013 and the final measurements in the last cohort (cohort 5) taken in March 2016.

#### This Study

Cohort 5 had 128 participants offered the e-message intervention during their 6-month class; 97 participants provided consent. Designed as an SMS text message study, to maximize inclusivity and allow participant preference, we offered an email-delivery option.

### Electronic Message Development

Verbal and written feedback from surveys and focus groups with participants in the first 4 cohorts of the study voiced concern that during months 7 to 12, when the class meeting frequency dropped to monthly, there was a feeling of decreased experimenter support and inadequate participant accountability. Decreased 7- to 12-month class attendance and diet adherence supported these subjective accounts. This e-message protocol enhancement was designed in response to this to increase class attendance and diet adherence for both LC and LF groups in cohort 5.

The e-message intervention was grounded in the nudge framework, whereby small touches can elicit significant behavior changes [18]. At a high level, receiving the SMS text or email message provides a cue to the treatment, a reminder that the participant is still in active treatment even though study interaction had decreased in frequency. More specifically, the questions elicited awareness of (1) the discrepancy between participants’ current behavior and their goal behavior and (2) their emotional response and subsequent coping behavior.

The first question, “how adherent have you been to your eating plan since your last survey?”, was based on the cybernetic model of self-control, which suggests that monitoring for discrepancies between the goal and current behavior can trigger behavioral corrections to mitigate the gap [19]. The subsequent 3 questions were based on feedback from the health coaches who taught the diet classes to participants in cohorts 1 to 4. They noted that many of the participants struggled with emotional eating and reported that discrepancy between goal and state increased the likelihood of the *what the hell* effect [20]. Research indicates that increased attention to emotional state is associated with a decreased emotional eating response [21]. Therefore, Question 2 showed a 7-point Likert scale depicting 7 faces from extreme negative to extreme positive emotion, with the text: “Based on the images below, how are you feeling about your adherence to your eating plan? Please click on the appropriate image.” Question 3 asked “What words would you use to describe your current feelings about your eating plan?” The possible responses were selected to fit in a 3×2 organizational structure of emotional responses to one’s goal actions: temporality (prospective, current, and retrospective) by valence (positive and negative) *.* Finally, Question 4 asked the participant’s behavioral response to the current emotion: “Given your current feelings and current eating plan adherence, which of the following actions are you motivated to do, if anything?” (see Multimedia Appendix 1). The responses to the e-messages are beyond the scope of this efficacy-only analysis. Importantly, though back-and-forth interactions were not a feature, soliciting responses to the questions from the participant made the SMS text messages 2-way, rather than 1-way pushes.

### Electronic Message Procedure

All consenting cohort 5 study participants who opted to receive the e-message intervention received 1 text or email with a link to a REDCap survey of 4 questions sent 3 to 4 times per week on randomly selected days each week [22]. Message frequency was based on Spark et al’s average contacts, with the goal of having regular, but not overwhelming, contact [16]. If the participant did not respond after 6 to 8 hours, 1 reminder was sent. Participants were encouraged to reply to each question, and each response was recorded within REDCap.

### Measures

Attendance was recorded for all 22 assigned classes. Weight was collected at baseline, 3 months, 6 months, and 12 months. Demographics such as age, gender, and race were collected at baseline. E-message responses were collected after the introduction at the 6-month mark. In this paper, we focus on 3 key outcomes: (1) proportion of class attendance at months 7 to 12; (2) diet adherence measured by 3 unannounced 24-hour recalls, as average grams of either total carbohydrate (LC) or total fat (LF) at 12 months, target being lower than matched controls total grams; and (3) weight change from 6 to 12 months.

As the assigned diets were to healthfully “go as low as you can sustain” in grams of either carbohydrates or fat, dietary adherence was defined by reduction of grams of the target diet. Dietary intake was assessed by using 3 unannounced 24-hour dietary recalls at each of the 4 major data collection time points throughout the yearlong study. Data were collected using the Nutrition Database System for Research, a computer-based software application developed at the University of Minnesota Nutrition Coordinating Center. Data were collected using a standardized multiple-pass interview approach to increase accuracy. Average daily grams of carbohydrates and fats were calculated from the 3 24-hour diet recalls. Even though there is bias in 24-hour recalls, because food records potentially have reactivity bias, the 24-hour recall is considered the least biased of the self-reported instruments and the best single dietary assessment instrument for many purposes [23]. Using 2 weekdays and 1 weekend day and not announcing the assessment days are methods of minimizing some error [23]. For more details, consult the separate methods paper for this study [17].

In addition, within the cohort 5 group that consented to participate, the relationship between the percentage of e-messages responded to (engagement) and all 3 outcomes mentioned in the previous paragraph was investigated.

Two questions were given at the end of the intervention for participants to evaluate the perceived usefulness of the e-messages and their preferred frequency of e-message receipt. Perceived usefulness was measured on a 5-point Likert scale with labels: 1, *not at all useful*; 2, *slightly useful*; 3, *somewhat useful*; 4, *moderately useful*; and 5, *extremely useful*. Preferred e-message frequency had 6 response choices. Of them, 4 response choices were in decreasing frequency of messages per week: *6-7*, *4-5*, *2-3*, or *1*. *Not sure* and *text messages did not help* were the final response choices. Open-text space was provided for intervention comments or improvement suggestions.

### Study Design

This was a longitudinal observational study of those who opted to participate in the e-message intervention in cohort 5, with a comparison with their matched historical controls from cohorts 1 to 4.

### Statistical Analysis

Propensity score matching can control for observed potential confounding covariates [24,25]. A propensity score model was used to generate a matched control group for the e-message participants in cohort 5 [26]. The propensity score model was developed using only cohort 5 data and capitalizes on cohort 5 having both participants who selected to be in the e-message study (n=97) and participants who were offered but chose not to participate (n=31). The propensity score model included the following covariates: age at 6 months, sex, race, median-centered weight change before 6 months, median-centered proportion of attendance before 6 months, and text consent. The propensity score model was estimated using data from cohort 5 only as cohorts 1 to 4 did not have the opportunity to refuse consent to the e-message intervention.

Participants in cohort 5 who selected to participate in the e-message intervention were matched 1:1 to individuals in cohorts 1 to 4 using the following variables: propensity score (P-score: propensity to participate or not, calculation described above), age, sex, race, proportion of classes attended before 6 months, and weight change before 6 months [27]. Absolute standardized differences between the e-message group and the controls were checked both pre- and postmatch to ensure balance was achieved by the matching procedure. The matching procedure generated a control group comparable with the treatment group on potential confounding variables, allowing us to attribute differences in our outcomes to treatment (e-message or control), assuming no unmeasured confounders. Matching was executed using the R package *optmatch* [27]. After generating the control group, a 2-sample *t* test was used to assess the differences between the e-message group and the control group in the 2 primary study outcomes (attendance and diet adherence) and 1 secondary outcome (weight loss maintenance). Diet adherence was measured by grams of fat for LF and grams of carbohydrates for LC, and consequently, adherence was analyzed within each diet as the ranges of fat and carbohydrate could not easily be compared (eg, fat on the order of 60 g and carbohydrate on the order of 130 g).

Scatterplots and Spearman correlations were used to assess the relationship between the proportion of e-messages responded to and the 3 outcomes described in a previous section: (1) proportion of class attendance after 6 months, (2) diet adherence measured as average total carbohydrate grams for LC and average total fat grams for LF at 12 months, (3) 12-month minus 6-month weight change. All statistical analyses were performed using R version 3.3.3 [28].

## Results

### Method of Message Delivery

Of the 88 participants with complete data for the analyses, 46 chose SMS delivery, 36 chose email, and 6 chose both. No differences in the primary outcomes were found by the method of participation (see Multimedia Appendix 2). Therefore, we collapsed the groups for the analyses.

### Baseline Characteristics

Baseline demographics such as age, gender, race, weight change before 6 months, and proportion of class attendance before 6 months are displayed by intervention and historical control group in Table 1 *,* along with estimated propensity scores (P-scores), both pre- and postmatch. Prematch and postmatch adjusted means, standardized differences, and *P* values were also included to compare the balance between the intervention and control groups before and after the match. Control group before the matching refers to all of cohorts 1 to 4 with sufficient data for the match (n=376); control group postmatch was 1:1 nearest neighbor matched to cohort 5 e-message participants with no missing weight data at 6 months (n=88).

The mean age for the intervention group was 39.5 years, whereas the mean age for the control group was 41.2 and 39.6 years pre- and postmatch, respectively. Similarly, the mean proportion of attendance before 6 months in the intervention group was 79.4, whereas the mean proportion of attendance before 6 months was 81.8 and 80.3 pre- and postmatch, respectively. The standardized differences postmatch for the intervention and control groups were closer to 0 than those for the prematch group, and all passed the rule of thumb of 0.2 for small effect sizes [29].

**Figure 1:**
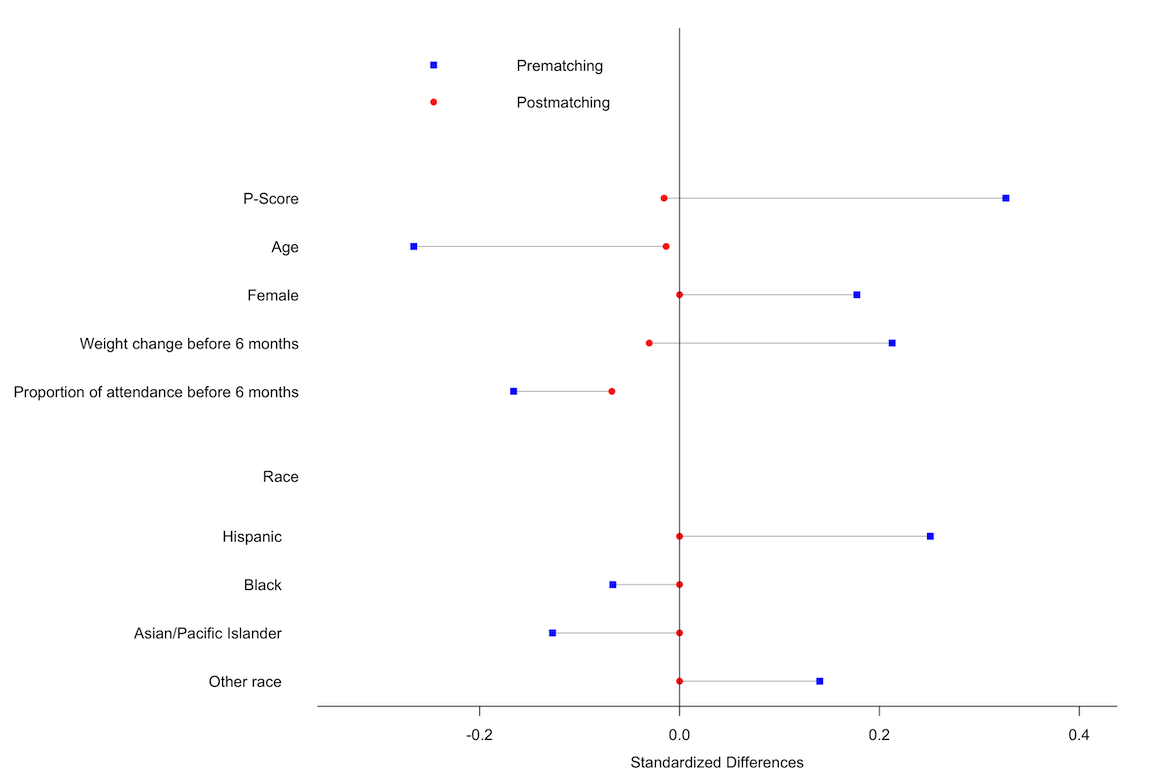
Standardized differences in study variables in electronic message group vs control group pre- and postmatch.

Standardized differences postmatch were 0 for gender and race and −0.02, −0.01, −0.03, and −0.07 for P-score, age, weight change before 6 months, and proportion of class attendance before 6 months, respectively. Figure 1 shows the balance between intervention and control pre- and postmatch. P-score, age, and Hispanic ethnicity were found to be significant prematch, but all variables were nonsignificant postmatch. As these results indicated that postmatch intervention and control groups were better balanced, the study hypotheses were only tested using postmatch groups.

### Effectiveness of Electronic Message Intervention

No statistically significant differences were seen in proportion of class attendance after 6 months between participants who received the e-message intervention (mean 57.0, SD 29.6) and those who did not (mean 52.4, SD 31.2); mean difference of intervention minus control was 4.6 (95% CI −4.4 to 13.7, *P*=.31; see Table 2). No significant differences were seen in diet adherence (total grams of carbohydrate per day) between participants on the LC diet in the intervention group (mean 133.7, SD 49.2, n=43) and those on the LC diet in the control group (mean 136.2, SD 63.3, n=39); mean difference was −2.5 (95% CI −29.9 to 24.8, *P*=.85). Similar results were observed for participants in the LF diet (grams of fat per day) between the intervention (mean 63.6, SD 23.4, n=45) and control groups (mean 57.4, SD 26.8, n=49); mean difference was +6.2 (95% CI −4.6 to 17.0, *P*=.26). No significant differences were seen in weight change between participants who received the intervention (mean 2.0, SD 4.3) and the control group (mean 2.0, SD 3.4); mean difference was 0.3 (95% CI −1.0 to 1.5, *P*=.68; see Table 2).

**Table 1:** Descriptive statistics for study variables in the electronic message group versus the control group pre- and postmatch. Means reported for age, weight change, class attendance; proportions reported for sex and race. SD=standardized difference (unitless measure of similarity, where closer to 0 is more similar). Variables with blank SD were not included in the match.

Variable		Prematch				Postmatch			
		Control (n=376)	Intervention (n=88)	SD	*P* value	Control (n=88)	Intervention (n=88)	SD	*P* value
Age (years)		41.2	39.5	−0.27	.03	39.6	39.5	−0.01	.70
Female (prop^a^)		0.57	0.66	0.18	.13	0.66	0.66	0	>.99
Hispanic (prop)		0.2	0.3	0.25	.03	0.3	0.3	0	>.99
Black (prop)		0.03	0.02	−0.07	.57	0.02	0.02	0	>.99
Asian/Pacific Islander (prop)		0.12	0.08	−0.13	.28	0.08	0.08	0	>.99
Other (prop)		0.05	0.08	0.14	.24	0.08	0.08	0	>.99
Weight (kg) change 6 months		−7.5	−6.2	0.21	.07	−6.0	−6.2	−0.03	.50
Class attendance 6 months (%)		81.8	79.4	−0.17	.16	80.4	79.4	−0.07	.15
Propensity score		0.84	0.86	0.33	.01	0.87	0.86	−0.02	.26
**Adherence at 6 months**									
	Low carb (g)	112.8^b^	115.9^c^	—^d^	—	114.9^e^	115.9^c^	—	—
	Low fat (g)	217.2^f^	206.7^g^	—	—	217.1^h^	206.7^g^	—	—

^a^prop: proportions.

^b^n=195.

^c^n=43.

^d^Not applicable.

^e^n=39.

^f^n=181.

^g^n=45.

^h^n=49.

**Table 2:** Estimated means and mean difference per outcome in the electronic message group versus the control group.

Outcome		Electronic message (n=88), mean (SD)	Control (n=88), mean (SD)	Mean difference	95% CI	*P* value
Class attendance		57.0 (29.6)	52.4 (31.2)	4.6	−4.4 to 13.7	.31
**Diet adherence (average g/day at 12 months)**						
	Low carb (n=82)	133.7 (49.2)^a^	136.2 (63.3)^b^	−2.5	−29.9 to 24.8	.85
	Low fat (n=94)	63.6 (23.44)^c^	57.43 (26.8)^d^	6.2	−4.1 to 17.0	.26
Weight change (kg)		1.9 (4.3)	1.6 (3.4)	0.3	−1.0 to 1.5	.68

^a^n=43.

^b^n=39.

^c^n=45.

^d^n=49.

### Electronic Message Engagement Within Intervention Participants

E-message response rate changed over time. Overall, 64 out of the 97 participants who received the intervention responded to e-messages during the first 2 weeks, whereas only 29 responded by the last week of the intervention. The average proportion of e-messages responded to out of those received by each participant (6-8 messages per 2-week period) started at 77% and declined to 25% by the end of the 6-month period (see Multimedia Appendix 3).

**Figure 2:**
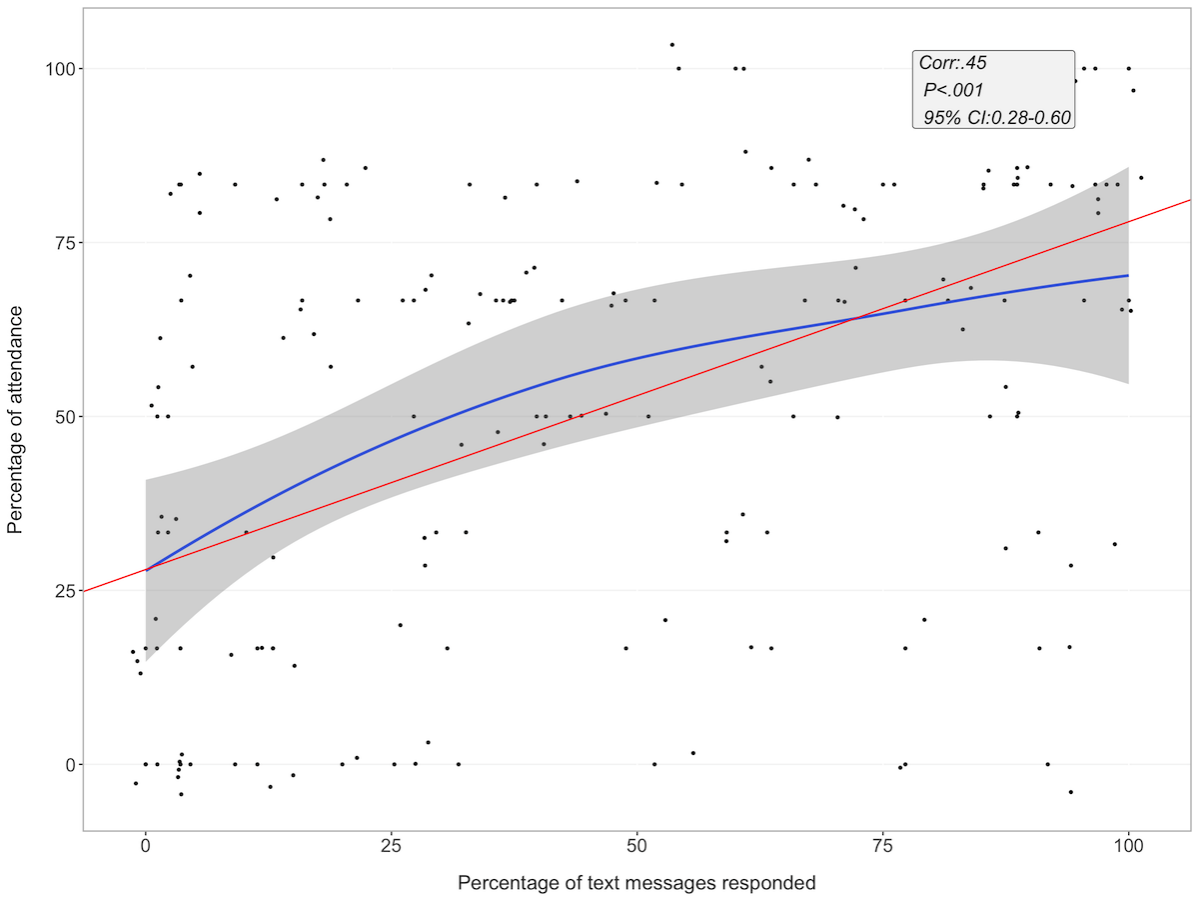
Electronic message response rate and class attendance after 6 months. Blue line denotes loess line fit and red line denotes linear regression line fit. E-message: electronic message; Corr: correlation.

The percentage of people who responded to at least one message per week began at 94% (63 out of 67 participants) and ended at 38% (37 out of 67 participants; see Multimedia Appendix 4). There was a positive relationship between e-messages and percentage of classes attended (*r*=.45, *P*<.001; see Figure 2).

There were no significant relationships between e-message response rate and diet adherence (*r*=−.03, *P*=.87) or e-message response rate and weight change (*r*=−.09, *P*=.41).

### Participant Perceptions of Electronic Messages

Perceived usefulness of the e-message intervention was answered by 58 of the 88 participants. On a 5-point Likert scale of perceived usefulness, 18 of them reported *not at all*; 28, *slightly useful*; 11, *somewhat useful*; 1, *moderately useful*; and 0, *extremely useful*. When asked about the preferred frequency of e-messages, 3 selected 6 to 7 messages/week, 5 chose 4 to 5 messages/week, 15 chose 2 to 3 messages/week, 9 chose 1 text/week, 5 chose not sure, and 21 said the e-messages did not help.

## Discussion

### Principal Findings

In response to a request from previous participants desiring more accountability for adhering to their diet, a low-cost e-message intervention was offered to the final cohort. Receiving frequent e-message prompts during the second 6-month period of the diet intervention to monitor goal adherence, emotional responses, and behavioral responses to goal discrepancies did not significantly improve class attendance, diet adherence, or weight loss retention compared with matched controls. Within those who did receive the e-messages, there was a positive relationship between overall e-messages response rate and classes attended during the second 6-month period; however, no relationship to diet adherence or weight change was found. One explanation for this association is that response to the e-messages is a proxy of program engagement, reflecting the action of participants already engaged.

One possibility for the null result is insufficient engagement in the e-message intervention. There was a significant reduction in e-message responses over time, with 75% of participants responding to any e-messages by week 5 and less than half of the participants responding to 1 e-message per week by the end of the intervention. Moreover, 18 of the 58 respondents who answered the postintervention questionnaires rated the messages as not useful at all, and when asked how frequently they thought the messages should be sent, 21 said the e-messages did not help. Almost half the participants (40/88) made open-ended suggestions and comments for improvement of the e-messages. Coauthors categorized these responses into broad themes of suggestions. Some of these themes were more variability of questions and possible responses (n=8), reducing the frequency of messages (n=6), more adaptable and accountable e-messages (n=5), messages were “annoying” (n=4), and response choices for “what words would you use to describe your current feelings about your eating plan?” were too ambiguous (n=4). Only 5 of the 40 who gave comments reported that it worked for them. Future e-message interventions may increase engagement by varying the message questions or content, thus decreasing possible message fatigue.

### Limitations

The study had several limitations. Although our e-messages elicited a participant response designed to increase the participant’s awareness of their diet adherence and internal reaction to it, there was no feedback, tracking of progress, or even automatic reply sent when the participant did respond. To increase participant engagement in the e-message intervention, a 2-way interaction could be provided. A recent review of self-directed weight loss interventions suggested that individualized feedback, email counseling, and online social support seemed to enhance effectiveness [30]. Indeed, studies that have found significant effects on weight loss behaviors used several of these elements as well as customized content around behavioral tips [2,14,31,32]. Our e-message intervention had none of these features and consisted instead of 1-way pushed questionnaires that were designed to make participants more aware of their dietary progress and internal emotional states. It is possible that some of these features from past studies are critical to motivate behavior change. Indeed, Spark et al’s study had more *human* elements with experimenter-automated replies using the participant’s name and signing the assigned health coach’s name [16]. Even if automated, there is evidence that the *mere belief* of social presence enhances arousal and engagement [33]. Another limitation is that although propensity score adjustment for treatment selection bias strengthens the causal interpretations of our findings, this adjustment cannot balance across all possible confounders, only the ones we included. A final limitation is the low power because of our small sample size. The historically matched controls allowed for us to fully use cohort 5 for the intervention population; however, a larger sample size would have had more power to detect effects and would have allowed for more stratified subgroup analyses.

A unique strength of this study was using propensity score–matched historical controls to compare the e-message intervention’s effect on outcomes. In addition, the study provides data on potential limitations and what did not work as well as open-ended participant feedback on why. This material can inform the development of other treatment adherence interventions, especially those involving SMS text or email messages. Finally, optimizing content and frequency of *nudge* mobile interventions to promote accountability with minimal experimenter cost is an iterative process that requires multiple studies to identify the optimal method and frequency of participant contact. Using SMS text message offers a broader potential impact on public health across the socioeconomic and geographic spectrum [11].

### Conclusions

This e-message intervention did not have a main effect on treatment adherence measured by class attendance or diet adherence; however, it did indicate a measure of engagement, with a relationship between e-message response and class attendance. Despite the limited effect of e-messages, this work significantly contributes to the space of mobile health interventions and hopefully inspires adaptive trial design. Feedback from earlier cohorts requested more accountability, and these messages were a low-cost, automated tool to support this request. By focusing on minimal *nudge-like* interventions that are able to produce the most change with the least effort, it may be possible to generate powerful behavioral public health tools that can be extended to individuals across the socioeconomic spectrum. Those most in need of public health interventions often have the least capability to access resources. Continued efforts to develop these types of innovative interventions may help bridge the gap.

## Appendix

Multimedia Appendix 1 Eating plan adherence questionnaire.

Multimedia Appendix 2 Outcomes by method of participation.

Multimedia Appendix 3 Mean proportion electronic message response over time.

Multimedia Appendix 4 Percentage participants with greater than 1 response/week.

Multimedia Appendix 5 CONSORT‐EHEALTH checklist (V 1.6.1).

## Abbreviations

e-message: electronic message
DIETFITS: Diet Intervention Examining The Factors Interacting with Treatment Success
LC: low-carbohydrate
LF: low-fat
RCT: randomized controlled trial
SMS: short message service
