# Supplementary figures and images for: To Text or Not to Text: Electronic Message Intervention to Improve Treatment Adherence Versus Matched Historical Controls
Source: JMIR Mhealth Uhealth. 2019 Apr 9;7(4):e11720. doi: 10.2196/11720 (PMC6534047; doi:10.2196/11720)

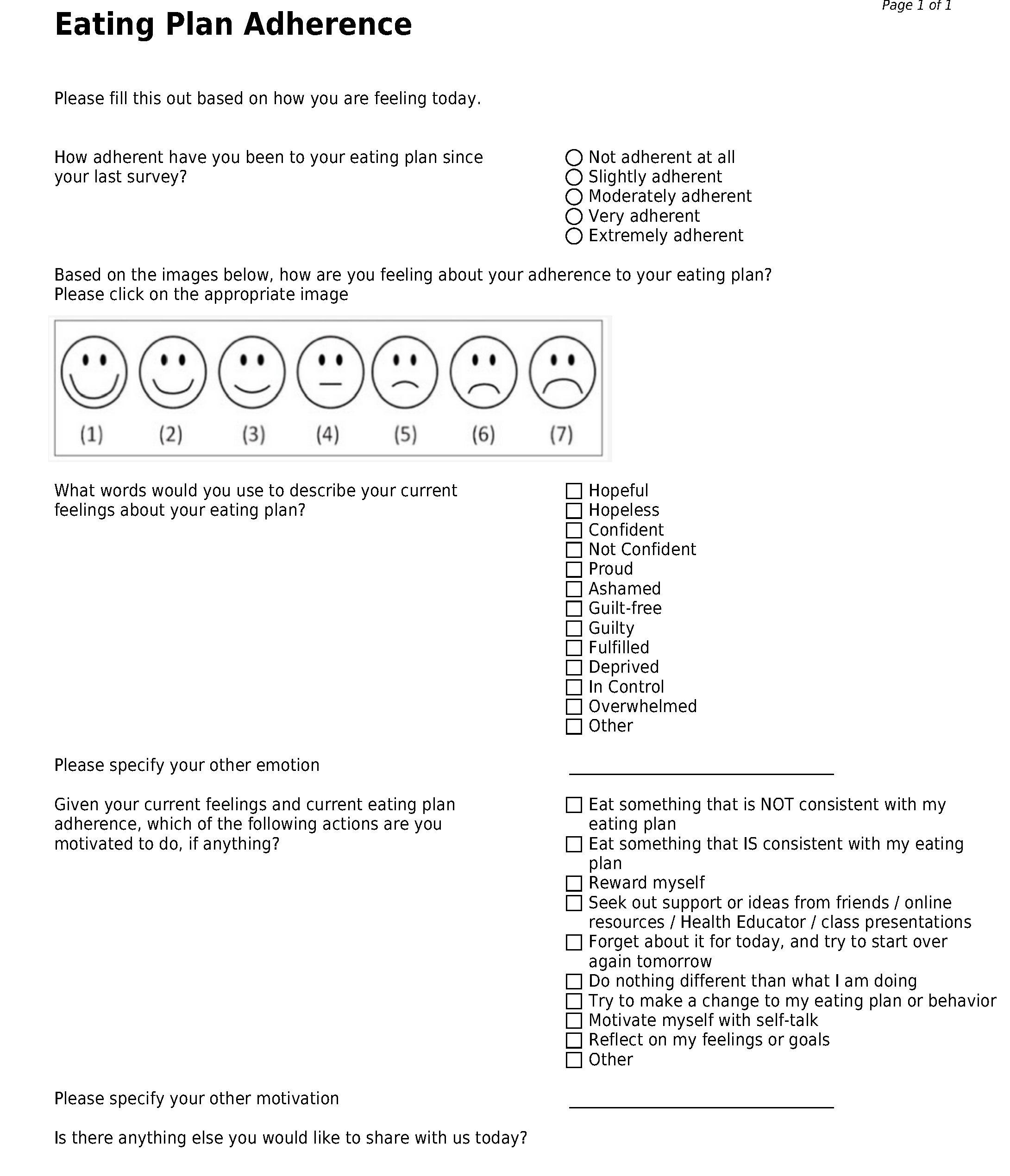

Supplement: Multimedia Appendix 1 [file mhealth_v7i4e11720_app1.png]

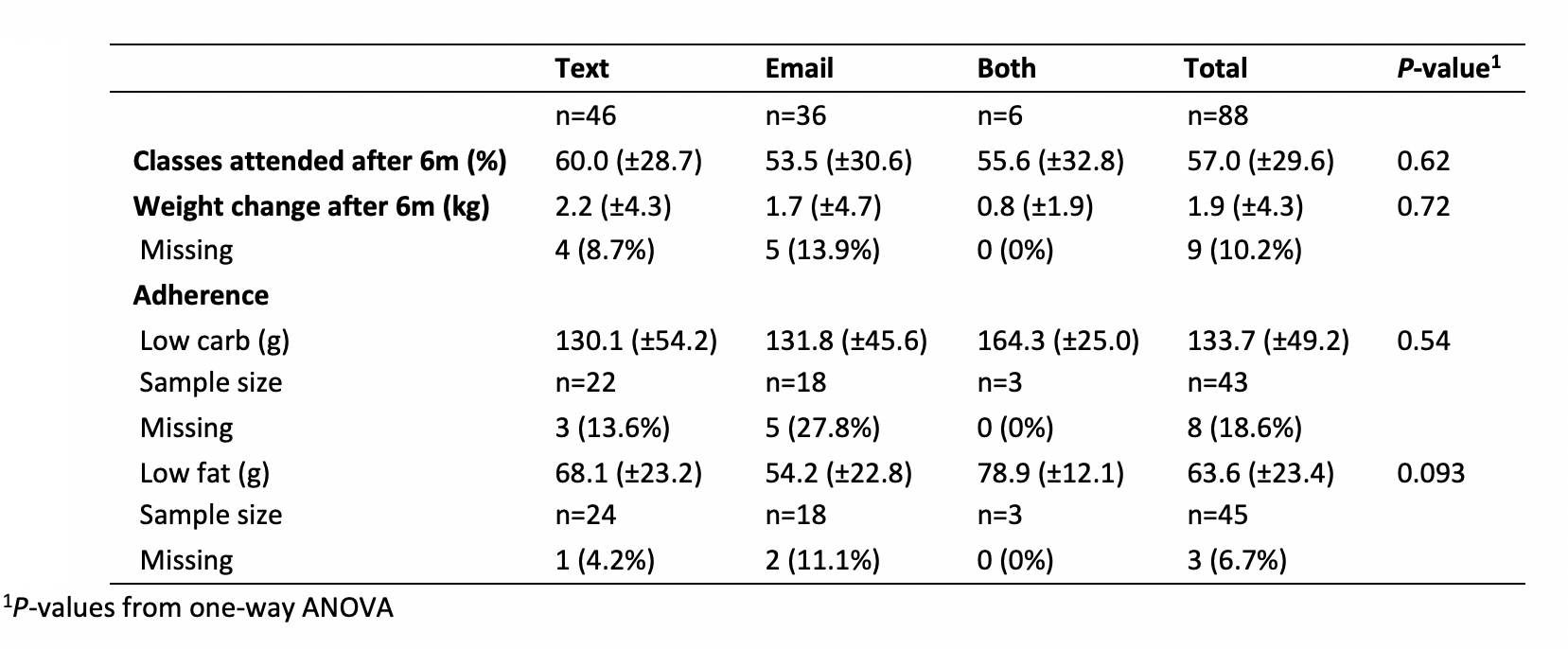

Supplement: Multimedia Appendix 2 [file mhealth_v7i4e11720_app2.png]

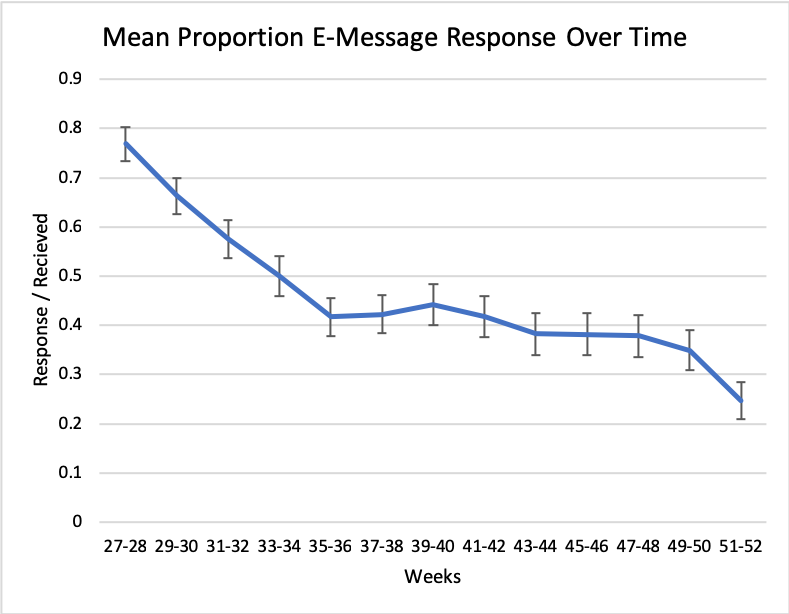

Supplement: Multimedia Appendix 3 [file mhealth_v7i4e11720_app3.png]

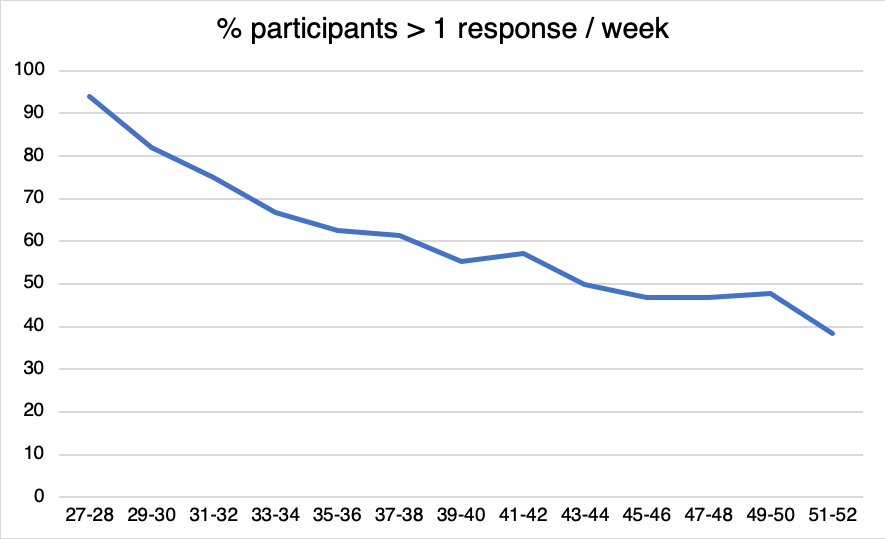

Supplement: Multimedia Appendix 4 [file mhealth_v7i4e11720_app4.png]
